# Roles of Zinc Signaling in the Immune System

**DOI:** 10.1155/2016/6762343

**Published:** 2016-10-31

**Authors:** Shintaro Hojyo, Toshiyuki Fukada

**Affiliations:** ^1^Osteoimmunology, Deutsches Rheuma-Forschungszentrum, Berlin, Germany; ^2^Faculty of Pharmaceutical Sciences, Tokushima Bunri University, Tokushima, Japan

## Abstract

Zinc (Zn) is an essential micronutrient for basic cell activities such as cell growth, differentiation, and survival. Zn deficiency depresses both innate and adaptive immune responses. However, the precise physiological mechanisms of the Zn-mediated regulation of the immune system have been largely unclear. Zn homeostasis is tightly controlled by the coordinated activity of Zn transporters and metallothioneins, which regulate the transport, distribution, and storage of Zn. There is growing evidence that Zn behaves like a signaling molecule, facilitating the transduction of a variety of signaling cascades in response to extracellular stimuli. In this review, we highlight the emerging functional roles of Zn and Zn transporters in immunity, focusing on how crosstalk between Zn and immune-related signaling guides the normal development and function of immune cells.

## 1. Introduction

In the 1960s, Prasad and his colleagues reported that zinc (Zn) plays indispensable roles in such diverse cellular events as cell proliferation, differentiation, and survival in humans [1]. In the past decade, studies in animal models have provided considerable knowledge about the molecular principles of Zn's function in the immune system, including new insights into how a single nutritional deficiency can alter immune-cell homeostasis and functions in both innate and adaptive immunity. Zn's physiological importance is supported by recent* in silico *studies showing that the proteins encoded by approximately 10% of entire genome in* Homo sapiens* can potentially bind Zn through Zn-finger motifs, RING finger domains, LIM domains, and PHD domains [2], all of which are involved in basic cellular activities [3]. In the present era, approximately two billion people in developing countries suffer from Zn deficiency (ZnD), mainly due to malnutrition and manifest clinical characteristics of growth retardation and compromised immune systems [1, 4]. ZnD induces thymic atrophy and lymphopenia and depresses both innate and adaptive immune responses: it impairs phagocytosis, intracellular killing activity, and cytokine production by macrophages; host defense by neutrophils and natural killer cells; and the proliferation, cytokine production, and antibody secretion of T and B cells [5]. These outcomes, which appear to be mostly rooted in the dysregulation of basic cellular functions such as DNA replication, RNA transcription, and cell activation, proliferation, differentiation, and survival [6–11], result in an increased susceptibility to a wide range of infectious agents and a longer duration of infection [1, 5].

Zn homeostasis is tightly controlled by Zn transporters and metallothioneins [12]. Zn acts as a cofactor for proteins and affects the structural and catalytic functions of enzymes and transcription factors [12]. In addition to recent mechanistic investigations into Zn's functions, other recent studies using chemical or gene-targeting approaches have revealed Zn's role as a second messenger, similar to that of cyclic adenosine monophosphate (cAMP) and calcium, in Zn signaling axes mediated by specific channels or Zn transporters [12].

In this review, we describe recent discoveries about the role of Zn signaling in the immune system and along specific Zn transporter axes. We will particularly focus on the physiological importance of Zn signaling in dendritic cells (DCs), T cells, and B cells, major populations that are required for crosstalk between the innate and adaptive immune systems. Ongoing research in this field will improve our understanding of the physiological significance of this vital trace element.

## 2. Physiological Roles of Zn Transporters in Zn Homeostasis

**Figure 1:**
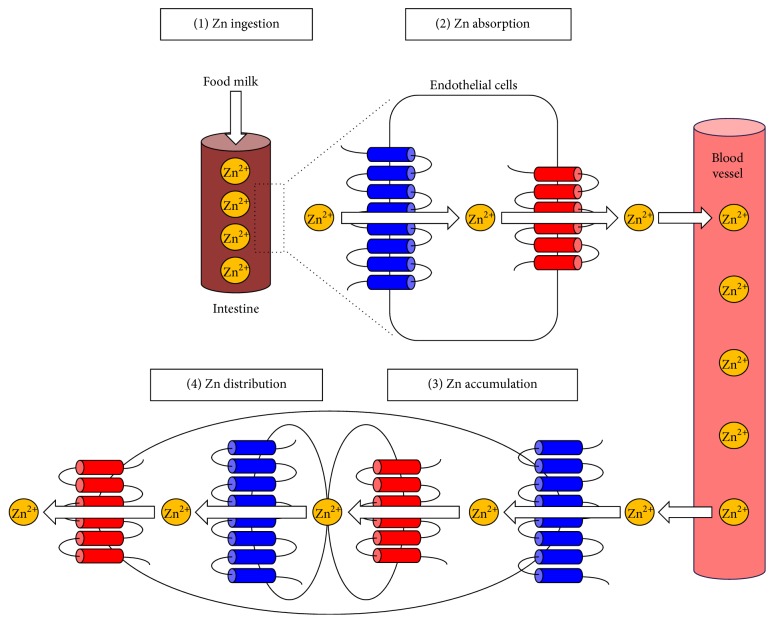
Zn transporters control Zn homeostasis. (1) Zn is ingested from the diet (or from breast milk in infants). (2) Zn is absorbed by intestinal Zn transporters and is released into the bloodstream. (3) Zn is taken up into peripheral cells by Zn transporters located on the plasma membrane. (4) Zn is distributed within the cell by intracellular Zn transporters. Each step is important for maintaining intracellular Zn levels. Disrupting Zn uptake results in ZnD and subsequent pathogenesis. ZIP (blue) and ZnT (red) transporters are indicated.

**Figure 2:**
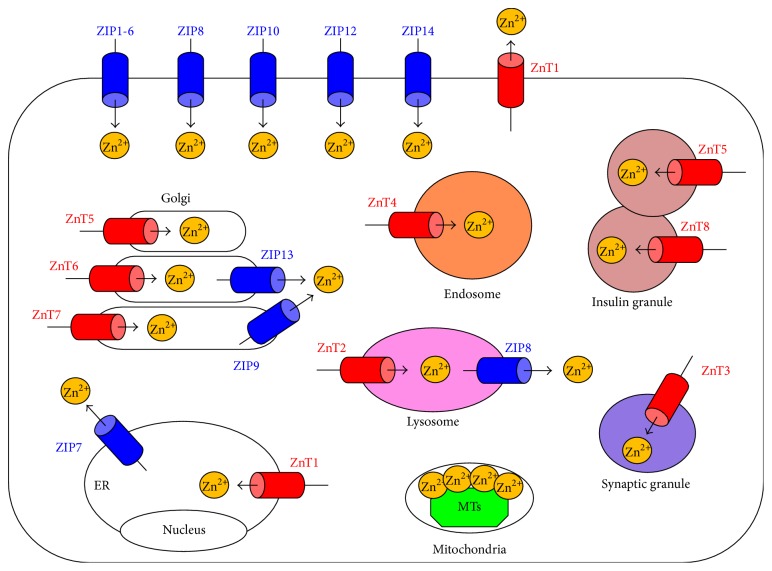
Subcellular localization of Zn transporters and metallothioneins (MTs). The localization of ZIP (blue) and ZnT (red) transporters is determined by the cell type, developmental process, and Zn status. ZIP transporters elevate the intracellular cytoplasmic Zn level by eliciting the influx of Zn from the extracellular space or from intracellular organelles. ZnT transporters reduce the intracellular cytoplasmic Zn level by exporting Zn from the cytosol to the extracellular space or into intracellular organelles or vesicles. MTs (green) contribute to Zn storage. Arrows indicate the predicted direction of Zn mobilization. ER: endoplasmic reticulum.

Zn is the second most abundant metal in the human body, with 2–4 grams distributed throughout the whole body. Zn is generally taken in through food or breast milk, is absorbed via several intestinal Zn transporters, and is released into the bloodstream (Figure 1). Circulating Zn is taken up into cells and distributed within the cell (Figure 1). At each step, Zn transporters and metallothioneins play coordinated roles in the transport, distribution, and homeostatic maintenance of Zn (Figure 2) [12]. Based on their predicted membrane topology, Zn transporters are divided into two major families, SLC39s/ZIPs and SLC30s/ZnTs, which mediate the inward and outward transport of Zn through cell-surface membranes and intracellular organelles (Figure 2) [13].

The ZIP family, which consists of 14 members containing eight putative transmembrane domains, elevates intracellular cytoplasmic Zn levels by eliciting the influx of Zn from the extracellular space or from intracellular organelles [12]. No ZIP crystal structure has been determined, but ZIPs are postulated to transport Zn by diffusion, symporters, or a secondary active transport system but not via an ATP-dependent mechanism.

Accumulated evidence from genetic approaches has demonstrated specific physiological roles of Zn transporters in mice and humans (Table 1) [12]. A single ablation of the ZIP1, ZIP2, or ZIP3 allele or a double ablation of ZIP1 and ZIP2 in mice leads to abnormal embryonic development in the ZnD environment of the mother [14–16]. ZIP4, the most intensively studied Zn transporter in human genetics, serves as a first gate for absorbing Zn into the body through the apical membrane of enterocytes [17]. Loss-of-function mutations in ZIP4 lead to acrodermatitis enteropathica (AE; OMIM 201100), a rare, pseudodominant, lethal genetic disorder characterized by severe ZnD symptoms such as periorificial and acral dermatitis, alopecia, and diarrhea in infants [17–20]. Supplementing with at least 1-2 mg Zn per kg of body weight per day dramatically improves the health and saves the lives of these patients, who would otherwise die within 2 years [17, 19]. In mice, targeted disruption of the ZIP4 gene impairs early embryonic development, since ZIP4 is thought to transport Zn into the embryo via the visceral yolk sac and later by the uptake of dietary Zn through the intestine [21]. Furthermore, mice with conditional ZIP4 ablation in enterocytes exhibit dramatic wasting and death unless they receive additional dietary Zn through nursing or supplementation [22]. These mice have reduced labile Zn and abnormal gene expression in Paneth cells, leading to an abnormal stem-cell niche in the crypts and subsequent disorganization of the absorptive epithelium. Loss-of-function ZIP5 mutations are associated with autosomal dominant nonsyndromic high myopia [23]. ZIP8-hypomorphic mice are embryonically lethal due to abnormal hematopoiesis and organ morphogenesis [24]. ZIP8 is also associated with human and murine osteoarthritis, in which an influx of Zn into cartilage chondrocytes elevates the expression of matrix-degrading enzymes [25]. Most recently, it has been reported that a nonsynonymous variant in ZIP8 is associated with schizophrenia [26]. A lack of ZIP10 impairs B-cell development and function in mice [27, 28]. Targeted ZIP12 disruption attenuates the development of pulmonary hypertension in rats in a hypoxic atmosphere [29]. ZIP13-deficient mice have abnormal systemic and bone growth with characteristics reminiscent of human Ehlers-Danlos syndrome (EDS), a group of recessive genetic disorders that affect connective tissue development, and of Osteogenesis Imperfecta, formally named as Spondylocheirodysplastic Ehlers-Danlos syndrome (SCD-EDS, OMIM 612350) [30–35]. ZIP14-deficient mice have impairments in systemic growth, bone metabolism, and gluconeogenesis [35], impaired liver regeneration (after partial hepatectomy) [36], decreased insulin signaling and hypertrophied adipocytes, and increased adipose cytokine production and plasma leptin [37]. In addition, it has been recently reported that the homozygous loss-of-function mutations of ZIP14 cause progressive parkinsonism-dystonia and neurodegeneration with hypermanganesemia in childhood [38].

The ZnT family, which consists of 10 members containing six transmembrane domains [12], reduces intracellular cytoplasmic Zn levels by exporting Zn from the cytosol to the extracellular space or into intracellular organelles or vesicles. Mice with a targeted disruption of ZnT1 exhibit embryonic lethality [39]. In mice, a targeted disruption or mutation of ZnT2 results in severe ZnD in nursing pups due to the extremely low Zn content of breast milk; a genetic mutation is known to cause the same symptoms in humans [40–43]. ZnT3 localizes to presynaptic vesicles, which are required for extracellular signal-regulated kinase (ERK) activation and hippocampus-dependent memory [44]. A targeted ZnT3 disruption causes memory deficits with Alzheimer's disease-like abnormalities in mice [45]. Similar to ZnT2-deficient mice, the milk of mice with a loss-of-function mutation in* ZnT4* (*lethal milk* mutant) is deficient in Zn, resulting in postnatal lethality [46]. ZnT5-deficient mice exhibit severe osteopenia, sudden death from bradyarrhythmia in males in their reproductive period [47], and impaired delayed-type allergic responses mediated by mast cells [48]. ZnT7-deficient mice show stunted growth due to a decrease in body fat accumulation [49]. In addition, ZnT7-deficient males are resistant to insulin and become hyperglycemic and glucose intolerant on a high-fat diet [50]. The ZnT8 transporter is associated with both type 1 [51] and type 2 diabetes mellitus [52]. ZnT8-deficient mice have impaired insulin secretion and crystal formation in diabetes mellitus [53–55] and rapidly clear insulin from the liver [56]. ZnT10 mutations cause parkinsonism and dystonia with hypermanganesemia, polycythemia, and chronic liver disease [57–59].

## 3. Zn-Signal Axes (Zn-Zn Transporter Signaling)

Neurons respond to exocytotic stimuli by releasing vesicular Zn into the surrounding milieu, where it is probably taken up into adjacent postsynaptic neurons and glial cells via Zn-permeable channels [65–68]. Thus, Zn acts as a neurotransmitter [69–71]. Zn also mimics the actions of hormones, growth factors, and cytokines [72] and regulates their functions by changing their structures via direct binding [73, 74]. These data suggest that Zn not only acts as an accessory factor for the function of various cellular components but also behaves as a signaling molecule, like calcium and cAMP [75, 76]. In fact, Zn acts through Zn channels and transporters to regulate a variety of signaling cascades mediated by hormone and growth factor receptors [77], cytokine receptors [28, 78, 79], toll-like receptors (TLRs) [61, 80], and antigen receptors [27, 48], so called “Zn signaling” [75].

**Figure 3:**
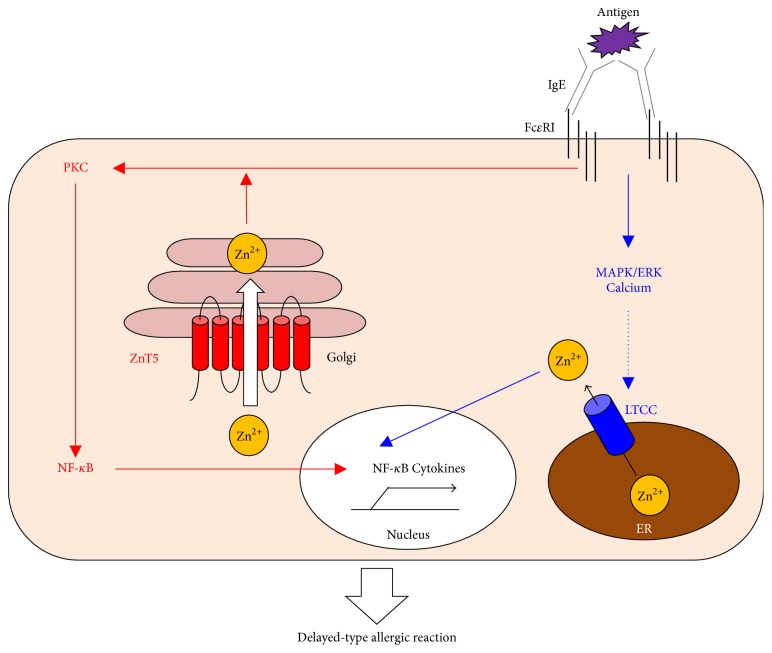
Zn uptake via LTCC and ZnT5 controls the Fc*ε*RI-mediated delayed allergic response by mast cells. Zn is indispensable for Fc*ε*RI-mediated mast-cell activation. Upon antigen sensitization, LTCC (blue) on the ER membrane acts as a Zn gatekeeper and can rapidly increase the intracellular-free Zn levels in the perinuclear region dependent on calcium and MAPK/ERK signaling (Zn wave). The released Zn then regulates the DNA-binding activity of NF-*κ*B followed by cytokine production. Simultaneously, ZnT5 (red) on the Golgi membrane takes up Zn to regulate the translocation of PKC to the plasma membrane. The resultant NF-*κ*B activation induces inflammatory cytokine production. Thus, these Zn gatekeepers and Zn transporter control mast-cell-mediated, delayed-type allergic reactions.

Zn's behavior as a second messenger has been clearly observed in some cell types. For example, mast cells, which induce allergic responses, express a surface Fc epsilon receptor I (FcɛRI) specific for IgE binding. Upon sensing an antigen through IgE-FcɛRI engagement, mast cells release intracellular granules containing histamine, lipids, and proteases to initiate an allergic response. Simultaneously, FcɛRI crosslinking induces a rapid release of intracellular Zn from the perinuclear area, including the endoplasmic reticulum (ER), in a phenomenon known as the Zn wave [81]. The Zn wave depends on calcium influx and mitogen-activated protein kinase (MAPK)/ERK activation and is mediated by the pore-forming *α*(1) subunit of the Cav1.3 (*α*(1D)) L-type calcium channel (LTCC) as a gatekeeper [82]. The LTCC-mediated Zn wave enhances NF-*κ*B's DNA-binding activity, inducing the gene transcription of inflammatory cytokines (Figure 3). In fact, LTCC antagonists inhibit the cytokine-mediated, delayed-type allergic reaction in mice without affecting the immediate-type allergic reaction [82]. Furthermore, Nishida et al. demonstrated using ZnT5-deficient mice that ZnT5 in mast cells plays a crucial role in the delayed-type allergic response represented by contact hypersensitivity (Figure 3) [48]. ZnT5 is highly expressed in mast cells and is upregulated by Fc*ε*RI stimulation. ZnT5-Zn signaling regulates the Fc*ε*RI-induced translocation of protein kinase C (PKC) to the plasma membrane, which induces NF-*κ*B activation, leading to the production of interleukin- (IL-) 6 and tumor necrosis factor alpha (TNF-*α*) [48]. Thus, Zn signaling via specific Zn channel and transporter controls FcɛRI-induced NF-*κ*B signaling in delayed-type allergic reactions (Figure 3).

Other instances of Zn signaling via Zn transporters have been observed. Zn transported by ZIP6 controls embryogenesis by regulating the nuclear translocation of SNAIL (a suppressor of E-cadherin transcription) during the epithelial mesenchymal transition (EMT) in zebrafish [79]. ZIP6-Zn signaling also negatively modulates the TLR-induced activation of DCs during immune responses [80]. The ZIP8-Zn axis negatively regulates NF-*κ*B activity by downmodulating the I*κ*B kinase activity in proinflammatory responses [61]. ZIP10-mediated Zn signaling suppresses caspase activity to promote cell survival in early B-cell development in the bone marrow [28] and regulates the activity of CD45R protein tyrosine phosphatase (PTPase) to control the strength of B-cell antigen receptor (BCR) signaling in antibody responses [27]. Most recently, Taylor et al. revealed that ZIP10 forms a heteromeric complex with ZIP6 and controls EMT through inactivation of GSK-3 and downregulation of E-cadherin in a breast cancer cell line and in zebrafish embryos [83]. Zn uptake mediated by ZIP13 and ZIP14 controls systemic growth and bone homeostasis; ZIP13 positively regulates SMAD's nuclear translocation in bone morphogenetic protein/transforming growth factor beta (BMP/TGF-*β*) signaling [34], and ZIP14 suppresses phosphodiesterase (PDE) activity to maintain cAMP levels in hormone-G-protein coupled receptor (GPCR) signaling [35]. ZIP14-induced Zn signaling also inhibits the protein tyrosine phosphatase 1B (PTP1B) activity, thereby increasing c-Met phosphorylation to promote hepatocyte proliferation during liver regeneration [36]. Collectively, these findings indicate that individual Zn transporters form specific Zn signaling axes to selectively organize distinct intracellular signaling events.

## 4. Roles of Zn and Zn Transporters in the Adaptive Immune System

ZnD's multifaceted effect on the immune system results in a high susceptibility to a variety of infections. Zn supplementation effectively improves immunity on the one hand and efficiently ameliorates chronic dysfunctional inflammatory responses on the other. These findings strongly suggest that Zn is essential for normal immune-cell homeostasis and function. There is already an excellent body of literature about Zn's roles in specific innate cell types, such as monocytes/macrophages and natural killer cells [84, 85], and we here focus on the roles of Zn and Zn transporters specifically in DCs, T cells, and B cells, which are bridging populations that enable crosstalk between the innate and adaptive immune systems.

### 4.1. DCs

DCs are professional antigen-presenting cells that are differentiated from a hematopoietic lineage and are important in linking the innate and adaptive immune systems. They circulate as immature cells and differentiate into mature DCs when activated by exposure to pathogens. In this process, DCs take up antigens, degrade them into peptides, load the antigenic peptides onto major histocompatibility complex II (MHC-II), and finally present the peptide-MHC-II complex on their cell surface to antigen-specific CD4^+^ helper T (Th) cells to initiate immune responses [86].

**Figure 4:**
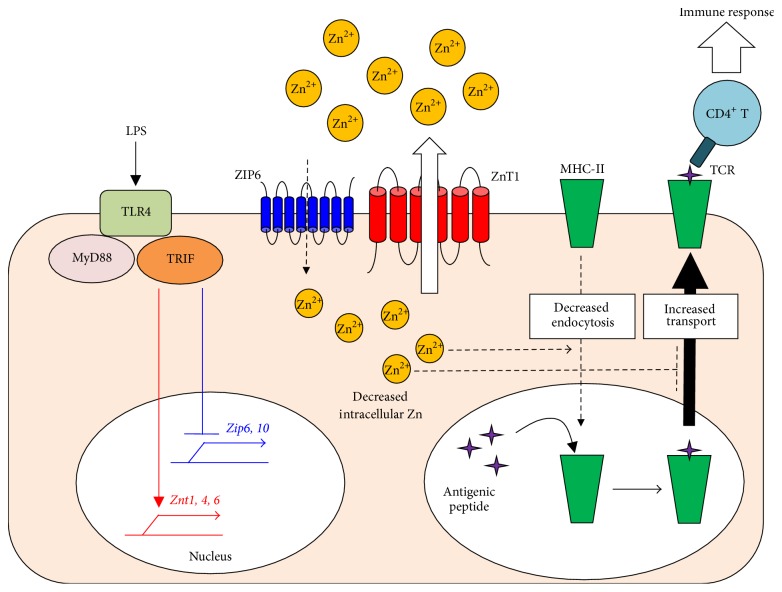
A decrease in intracellular-free Zn is critical for LPS-mediated CD4^+^ T-cell activation by DCs. LPS, a TLR4 ligand, induces DC activation, which initiates a maturation signal mediated by MyD88 and TRIF. TRIF-mediated signaling reduces the expression of ZIPs (blue) and increases that of ZnTs (red), resulting in a net decrease in the intracellular-free Zn level in DCs. This reduction of intracellular-free Zn is critical for the antigen presentation via MHC-II molecules and the subsequent activation of antigen-specific CD4^+^ T cells.

When mouse DCs* in vivo* or* in vitro* are exposed to a ligand for toll-like receptor 4 (TLR4), lipopolysaccharide (LPS), which is a component of bacterial cell walls, the DCs elicit cell maturation with a reduction in intracellular-free Zn levels and an increase in surface MHC-II and costimulatory molecules (Figure 4) [80]. During the maturation process, Zn transporter expression is changed: ZIP6 and ZIP10 are downregulated, while ZnT1, ZnT4, and ZnT6 are upregulated for a net decrease in cytosolic Zn content. Chemical Zn chelation by the membrane-permeable Zn chelator TPEN (*N,N,N,N* tetrakis (2-pyridylmethyl) ethylenediamine) mimics this phenomenon, while the forced introduction of Zn or the ectopic expression of ZIP6 suppresses LPS-induced DC maturation. Consistent with these findings, DCs that overexpress ZIP6 fail to activate antigen-specific CD4^+^ Th cells. Microscopic analysis revealed that Zn facilitates the endocytosis of MHC-II but inhibits the trafficking of MHC-II from the lysosome/endosome compartments to the plasma membrane. These results suggest that a reduction in cellular Zn is required for proper antigen presentation via MHC-II to elicit adaptive immune responses (Figure 4) [80].

Although ZnD causes immunodeficiency [1], it can also induce abnormal skin inflammations accompanied by erythematous rashes, scaly plaques, and ulcers on the acral and periorificial areas [17–20]. In fact, these paradoxical symptoms are particularly obvious in patients with hereditary and acquired AE, in which an immunostimulated skin inflammation develops in areas subject to repeated contact [19]. Interestingly, dietary ZnD mice with allergic contact dermatitis (ACD) induced by dinitrofluorobenzene (DNFB) show markedly reduced ear swelling, while those with irritant contact dermatitis (ICD) induced by croton oil (CrO) exhibit augmented ear swelling [60]. Histological analysis revealed that the ICD lesions in ZnD mice have features similar to cutaneous manifestations in human AE lesions, such as subcorneal vacuolization and epidermal pallor. In ZnD mice, damaged epidermal keratinocytes release adenosine 5′-triphosphate (ATP) that leads to ICD, which can be ameliorated by locally injecting soluble nucleoside triphosphate diphosphohydrolase. Notably, experiments in* ex vivo* organ culture showed that Zn chelation by TPEN enhances the ATP release in response to CrO whereas TPEN alone does not, suggesting that a combination of ZnD and chemical irritants synergistically increases the release of ATP from keratinocytes [60].

These findings raise the question of why a ZnD environment enhances the irritant-induced ATP release from keratinocytes. Langerhans cells (LCs) are epidermis-resident DCs that act as sentinels to orchestrate immune responses against foreign antigens, including pathogens, in the skin [87]. LCs exclusively express the ecto-NTPDase CD39 [88], which protects the cells against ATP-mediated inflammatory signals by hydrolyzing the extracellular nucleotides released by keratinocytes [88]. Interestingly, the number of epidermal LCs is significantly reduced in ZnD mice [60]. A similar phenomenon is observed in the lesions of patients with AE, but not those with atopic dermatitis or psoriasis vulgaris. Notably, LC recolonization of the epidermis and marked clinical improvement are observed in AE patients treated with oral Zn supplements. These data collectively suggest that inflammatory skin manifestations in AE patients may result from excessive ICD responses upon repeated exposure to various irritants in a ZnD environment, due to the aberrant ATP release from epidermal keratinocytes and the depletion of LCs [60].

### 4.2. T Cells

Activated T cells are largely divided into CD4^+^ Th cells which provide vital assistance to B cells to induce antibody response and CD8^+^ cytotoxic T cells (CTLs) which induce cell death by direct interactions with pathogen-infected cells or tumor cells. During an adaptive immune response, both types of cells establish an immunological memory that is poised to respond rapidly and effectively to a pathogen that has been previously encountered (recall response). Th-cell populations are involved in autoimmunity, allergic response, and tumor immunity. Upon T-cell receptor (TCR) activation in a particular cytokine milieu, naive CD4^+^ T cells can differentiate into several subsets, including Th1, Th2, Th17, and regulatory T (Treg) cells. The resultant CD4^+^ T-cell subsets are characterized by their functions and patterns of cytokine production [89]. Th1 cells promote cell-mediated immune responses against intracellular pathogens and produce the cytokines, interferon gamma (IFN-*γ*), TNF-*α*/*β*, and IL-2. These cytokines promote macrophage activation, nitric oxide production, and CTL proliferation, leading to the phagocytosis and destruction of microbial pathogens. The differentiation and expansion of Th1 cells are driven mainly by IL-12, which induces the signal transducers and activator of transcription (STAT) 4-dependent Th1-specific transcription factor T-bet. T-bet promotes the expression of IFN-*γ* and IL-12Rb2, which, together with IL-12Rb1, form a functional IL-12 receptor complex to further stimulate IFN-*γ*-induced Th1 differentiation. Exaggerated Th1 responses are associated with autoimmune diseases, including rheumatoid arthritis, multiple sclerosis, inflammatory bowel disease, and type 1 diabetes. Th2 cells, which are required for humoral immunity against extracellular pathogens, secrete IL-4, IL-5, IL-6, IL-10, and IL-13. Exposing TCR-stimulated CD4^+^ T cells to IL-4 induces the STAT6-dependent expression of the Th2 master transcriptional regulator GATA-3 and then produces IL-5 and IL-13 for cell expansion. IL-2, IL-7, or thymic stromal lymphopoietin (TSLP) is also required during Th2 differentiation to activate STAT5, which cooperates with GATA-3 to promote the T-cell production of IL-4. IL-4 regulates the clonal expansion of Th2 cells and, along with IL-13, promotes the B-cell production of IgE and alternative macrophage activation. Excessive Th2-type immune responses have been implicated in the development of chronic allergic inflammation and asthma. Th17 cells are involved in immune responses mounted against specific fungi and extracellular bacteria. In mice, Th17 cells develop from naive CD4^+^ T cells in the presence of TGF-*β* and IL-6, inducing the STAT3-dependent expression of IL-21, the IL-23 receptor, and the transcription factor ROR*γ*t. IL-21 and IL-23 regulate the establishment and clonal expansion of Th17 cells, while ROR*γ*t-induced gene expression leads to the secretion of IL-17A, IL-17F, and IL-22. Cytokines produced by Th17 cells stimulate resident cells to secrete chemokines to recruit neutrophils and macrophages to inflammation sites. The persistent secretion of Th17 cytokines promotes chronic inflammation and may be involved in the pathogenesis of inflammatory and autoimmune diseases such as rheumatoid arthritis, multiple sclerosis, and inflammatory bowel disorders. Tregs play an indispensable role in maintaining the immunological unresponsiveness to self-antigens and in suppressing excessive immune responses that would be deleterious to the host.

T cells reach maturity after passing through several stages in the thymus [90]. They begin as CD4^−^CD8^−^ double-negative (DN) thymocytes, pass through a double-positive (DP) stage (CD4^+^CD8^+^ thymocytes), become single-positive (SP) T cells (CD4^+^CD8^−^ or CD8^+^CD4^−^ SP thymocytes), and finally leave the thymus as naive T cells. In humans and mammalian model animals, ZnD causes thymic atrophy with a substantial reduction of DP thymocytes and a subsequent decline in mature T-cell counts [91–101]. Patients with AE due to a ZIP4 mutation show symptoms of severe ZnD characterized by immunodeficiency with thymic atrophy and lymphopenia and by recurrent infections in ~30% of patients [102]. One mechanism that contributes to this thymic atrophy is accelerated apoptosis, due in part to chronically elevated levels of glucocorticoids (corticosterone in particular) from the adrenal glands [103–108], although human thymocytes are relatively resistant to glucocorticoids [106]. In fact, adrenalectomized mice fed a ZnD diet show little change in thymic weight [105, 106], whereas, in adult mice given a corticosteroid implant, the thymus size is substantially reduced [109]. Furthermore, antiapoptotic proteins such as B-cell lymphoma- (BCL-) 2 and BCL-X are reduced in ZnD DP thymocytes, increasing their susceptibility to apoptosis compared to DN thymocytes, Th cells, and CTLs [110–112].* In vitro* studies showed that more lymphocytes and thymocytes undergo apoptosis when cultured in a Zn-free medium [113] or with a Zn chelator [114–116] as opposed to when cultured in a normal medium.

Another potential mechanism underlying thymic atrophy in a ZnD environment is the impaired activity of the nonapeptide thymulin (H-Pyr-Ala-Lys-Ser-Gln-Gly-Gly-Ser-Asn-OH) [117–119]. Thymulin, which is secreted by thymic epithelial populations, binds a specific high-affinity receptor on T cells to promote T-cell maturation, cytotoxic function, and IL-2 production [117, 118, 120–122]. Serum thymulin is present but less active in ZnD subjects; its activity is restored by Zn supplementation [117], suggesting that Zn promotes a conformational change in thymulin to confer biological activity [119]. Thus, the ZnD-induced thymic atrophy could result from the combination of increased glucocorticoid levels, an impairment of thymulin's activity and impaired cell-intrinsic survival function.

Which Zn transporter regulates early T-cell differentiation in the thymus is poorly understood. Mice with a targeted ZIP3 deletion have lower DP thymocyte counts and increased counts of CD4^+^ SP or CD8^+^ SP thymocytes under a Zn-limiting condition [14], suggesting that ZIP3 loss accelerates T-cell maturation. However, deleting ZIP3 does not change Zn homeostasis in terms of the levels of essential metals or the expression of Zn-responsive genes. These data suggest that ZIP3 plays an ancillary role in Zn homeostasis to generate naive T-cell populations in the thymus [14].

In contrast to the susceptibility of DP thymocytes to ZnD, mature CD4^+^ and CD8^+^ T cells are relatively resistant to ZnD and survive well in the atrophying thymus [112]. However, in adrenalectomized mice fed a ZnD diet, the Th-cell helper functions that promote the differentiation of B cells into antibody-secreting plasma cells are impaired, even though there is little change in the thymic weight [105, 106], indicating that Zn status is important not only in the early development of T cells but also in their activation and function in periphery. Indeed, microarray analysis showed that even a modest Zn deficiency in mice changes the expression of 1,200 genes related to the proliferation, survival, and response of T cells [123]. Furthermore, IL-2 production is decreased in mice with a marginal ZnD, even though there is no thymic shrinkage or increase in glucocorticoid concentrations [124, 125]. Several* in vitro* studies demonstrated that Zn is important for T-cell proliferation in response to cytokines and mitogenic agents [9, 126–132]. Zn is required during the mid to late G1 phase [10], the transition to S phase [133], and the transition to the G2 and M phases [134, 135]. Phytohemagglutinin- (PHA-) stimulated lymphocytes from mildly ZnD patients contain a greater proportion of cells at S phase than those from normal human controls; this increase is reversed by Zn supplementation [136].

TCR signaling is indispensable for cell proliferation, differentiation, and survival and for cytokine production [137]. Upon antigen recognition, the TCR stimulates LCK to activate the PI3K-AKT pathway and phosphorylate the immunoreceptor tyrosine-based activation motifs (ITAMs) of the TCR/CD3 complex on the cytosolic side, thereby recruiting and activating ZAP70, which in turn recruits and activates downstream adaptor or scaffold proteins such as SLP-76, VAV, and ITK. ITK activates phospholipase C, gamma 1 (PLC*γ*1), which leads to the production of the second messengers diacylglycerol (DAG) and inositol trisphosphate (IP3). DAG further activates the protein kinase C theta (PKC*θ*), NF-*κ*B, and MAPK/ERK pathways. On the other hand, IP3 induces the ER to release calcium. Calcium-bound calmodulin activates the phosphatase calcineurin (CN), which promotes IL-2 gene transcription through the nuclear translocation of transcription factor NFAT.

**Figure 5:**
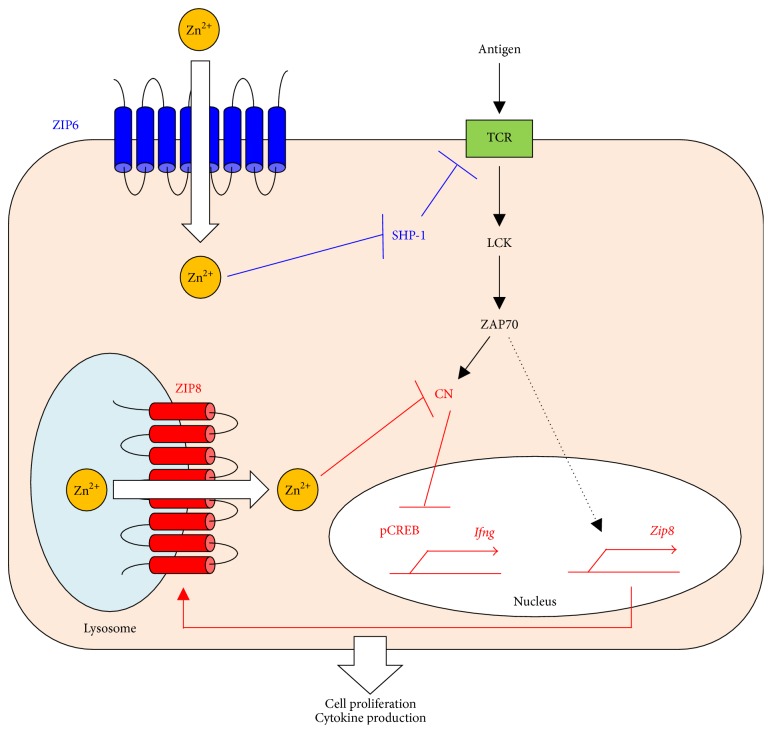
Zn uptake via ZIP6 and ZIP8 potentiates TCR signaling. Through the interaction of DCs and T cells, TCR activation rapidly increases cytoplasmic Zn concentrations, particularly at the subsynaptic compartment, in a manner dependent on ZIP6 (blue). The enhanced influx of Zn reduces SHP-1 recruitment to the TCR activation complex, thereby augmenting ZAP70 activation and leading to a sustained influx of calcium. On the other hand, TCR activation increases ZIP8 expression, which exports Zn out of the lysosome and into the cytoplasm (red). The resultant increase in cytoplasmic Zn inhibits CN, leading to increased CREB activation and the subsequent expression of IFN-*γ*. Thus, Zn facilitates TCR's functions in proliferation and IFN-*γ* production.

Zn is reported to affect components of the TCR signaling pathway [138, 139]. Some reports indicate that increased intracellular Zn concentrations enhance the activation of LCK and PKC [140] but inhibit the activity of the phosphatase CN [141, 142] and other PTPases. Stimulating T cells by incubation with DCs induces an influx of Zn across the plasma membrane via ZIP6, which rapidly increases the intracellular Zn concentration at spatially restricted regions; the Zn is concentrated near the immunological synapse between the T cell and DC [143]. This phenomenon enhances ZAP70 and inhibits the recruitment of tyrosine phosphatase SHP-1 (a negative regulator for TCR signaling) to the TCR, resulting in a prolonged calcium influx that contributes to cell proliferation and cytokine production (Figure 5). This mechanism suggests an important potential role for the Zn transporter-Zn signaling axis in TCR signaling.

ZIP8 is highly expressed in human T cells and is markedly upregulated by* in vitro* stimulation with TCR [144]. RNA interference against ZIP8 reduces the ZIP8 expression and attenuates the production of IFN-*γ* and perforin in human T cells. In contrast, overexpressing ZIP8 enhances the IFN-*γ* production. ZIP8 localizes to lysosomes, and labile Zn decreases in the lysosomes and increases in the cytoplasm during T-cell activation. Further analysis revealed that ZIP8-Zn reduces the CN phosphatase activity, leading to higher IFN-*γ* production following prolonged phosphorylation of the transcription factor CREB. Thus, ZIP8-Zn signaling positively controls TCR-induced cytokine production (Figure 5) [144]. In fact, several studies indicate that ZnD suppresses the production of cytokines such as IL-1, IL-2, IL-4, and IFN-*γ* [93, 145–152]. In Th0 and Th1 cell lines cultured in low Zn medium, mitogenic stimulation reduces the mRNA expression of IL-2 and IFN-*γ* [153]. In this context, experiments in humans revealed that Zn deprivation decreases the production of Th1 cytokines but has less effect on the production of Th2 cytokines (IL-4, IL-6, and IL-10), so the Th1/Th2 balance is disturbed and shifted toward Th2 [145, 149]. Consistent with this observation, cell-mediated immunity and delayed-type hypersensitivity fail in Th1 cells with a ZnD condition, whereas Th2-dependent function appears to be less affected [94, 145, 149, 154, 155]. In contrast, Zn enhances production of the Th1 cytokine IFN-*γ* and decreases the Th2 cytokine IL-10 in human PBMCs exposed to allergens [156]. These dysregulations associated with low Zn are restored by Zn supplementation [145, 149, 157–162], suggesting that Zn controls the Th1/Th2 balance.

Tregs play a special role in controlling immune homeostasis by suppressing undesirable immune responses [163]. Several reports indicate that Zn treatment prevents T-cell-mediated immune responses* in vivo* and* in vitro* [78, 164–166] and enhances the number and activity of Tregs in some cases [156, 167, 168].* In vitro*, Zn attenuates Th17 differentiation, which is controlled by IL-6-induced STAT3 activation during chronic inflammation by suppressing STAT3 activation [89]. Reflecting this result,* in vivo* studies show that moderate Zn treatment inhibits Th17-cell development and disease severity in mice with experimental autoimmune encephalomyelitis (EAE) and collagen-induced arthritis (CIA) [78, 167]. Moreover, at higher concentrations, Zn suppresses T-cell proliferation in mice [169] and cytokine production in human Jurkat [154] and CD4^+^ T cells [144]. Although the molecular mechanisms underlying these concentration-dependent effects of Zn is largely unknown, they may involve the overcapacity of the intracellular buffering system to absorb large amounts of Zn and a breakdown of the system of Zn transport. Thus, Zn immunomodulates cytokine signaling in T cells to control antigen-specific immune reactions.

Although many reports have revealed the importance of nutritional Zn in classical Th subsets, Zn's effect* in vivo* on follicular helper T (Tfh) cells, an important component in humoral immunity, has not yet been addressed. Tfh cells are a subset of specialized effector Th cells that help B cells and are essential for germinal center (GC) formation, which promotes the generation of high-affinity antibody-secreting plasma cells and memory B cells* in vivo* [170]. Although it is still controversial whether Tfh cells are differentiated from a lineage that is independent from those of Th1, Th2, Th17, and Treg cells, Tfh cells differentiate through multistage and multifactorial processes that accommodate significant heterogeneity. Tfh-cell differentiation begins with the priming of naive CD4^+^ T cells by DCs. Early Tfh cells can migrate to the border between the periarterial lymphatic sheath (PALS) and B-cell follicles; this migration depends on the CXCR5 chemokine receptor. Finally, the Tfh cells mature in B-cell follicles. Tfh cells express relatively high levels of BCL6, a critical regulator of Tfh differentiation, and secrete IL-4 and IL-21, which cause B cells to induce class-switch recombination (CSR) for immunoglobulin (Ig) (e.g., IgM to IgG1 and IgE) and to elicit GC persistence, respectively [170, 171]. IL-2 antagonizes Tfh differentiation in a T-cell-intrinsic manner, and IL-2 deficiency augments the generation of Tfh cells and enhances GC formation [172]. ZnD impairs the T-cell-induced IL-2 production [145, 149, 154] but also compromises GC formation, thereby decreasing the subsequent IgG1 production [27]; thus, it is possible that ZnD also affects the generation of Tfh cells. This idea is supported by the fact that BCL6 bears a C-terminal Zn cluster, consisting of six Zn-finger domains, that is necessary for its DNA binding to target genes [173]. Future studies should focus on clarifying Zn's role and identifying the Zn transporter involved in Tfh-cell generation in a variety of immunological contexts.

### 4.3. B Cells

B cells play crucial roles in the humoral immune response, which is a major weapon in the adaptive immune system [174, 175]. B cells develop initially in the bone marrow. Pro-B cells commit to pre-B cells followed by immature (IMM) B cells, which express BCR on their cell membrane. The IMM B cells then migrate to the spleen, where they further differentiate into transitional (TR) B cells and then into mature B-cell populations such as follicular (FO) and marginal zone (MZ) B cells.

Mature B cells, which are classified as professional antigen-presenting cells along with DCs, capture and process antigens taken up by specific BCRs, load antigenic peptide onto MHC-II, and present it to CD4^+^ Th cells. Among the mature B cells, FO B cells account for the majority of splenic B cells and are crucial for T-cell-dependent (TD) immune responses. During an immune response, activated FO B cells form the GC in the follicle with the help of Tfh cells, and they undergo massive expansion, with somatic hypermutation and CSR (e.g., IgM to IgG1) of the Ig genes to acquire a high-affinity Ig [176]. In contrast, MZ B cells, which are noncirculating, mediate rapid T-cell-independent (TI) immune responses against blood-borne pathogens.

Cook-Mills and Fraker indicate that ZnD has little effect on antibody secretion [177]. However, the plaque-forming cell (PFC) assays in this study were interpreted by evaluating the antibody-secreting ability of the surviving residual cells in ZnD mice on a per-cell basis not on a whole-organ scale. Other reports show that ZnD depresses both TD and TI antibody responses [96, 99, 178, 179]. In addition, ZnD reduces the TD antibody responses against sheep red blood cells by 90% of control [96, 125] and reduces the TI antibody responses against dextran by 50% of control [124, 180]. In contrast, both the IgM and IgG PFC activities are restored when ZnD mice are fed a normal diet, although the recovery of IgG PFC is greatly delayed [96, 178]. These observations strongly suggest that Zn controls antibody-mediated humoral immune responses.

The ZIP-family member ZIP10, which is a cell-membrane-localized transporter, is expressed in splenic B cells [27]. ZIP10 transports Zn from the extracellular fluid to the intracellular space [27, 28]. The targeted disruption of ZIP10 in antigen-presenting cells, including mature B cells, diminishes antigen-specific antibody responses, in particular, the production of IgG antibodies, which is correlated with severe GC-formation impairment in a B-cell-intrinsic manner [27]. Furthermore, in immunized mice fed a ZnD diet, the GC B-cell population and the antigen-specific IgG1 response are significantly reduced, partly mimicking the phenotypes in ZIP10-deficient mice. Although the number of mature resting FO B cells is significantly decreased in ZIP10-deficient mice* in vivo*, their proliferative activity in response to BCR stimulation is also reduced* in vitro*. Moreover, TI responses are also impaired in ZIP10-deficient mice, which have an intact number of MZ B cells. Collectively, these results suggest that Zn not only quantitatively controls FO B-cell maintenance but also qualitatively regulates the BCR signaling pathway. Thus, the abrogated TD antibody response in ZIP10-deficient mice cannot be fully explained by the reduced FO B-cell count; impaired BCR signaling is also appreciably involved.

**Figure 6:**
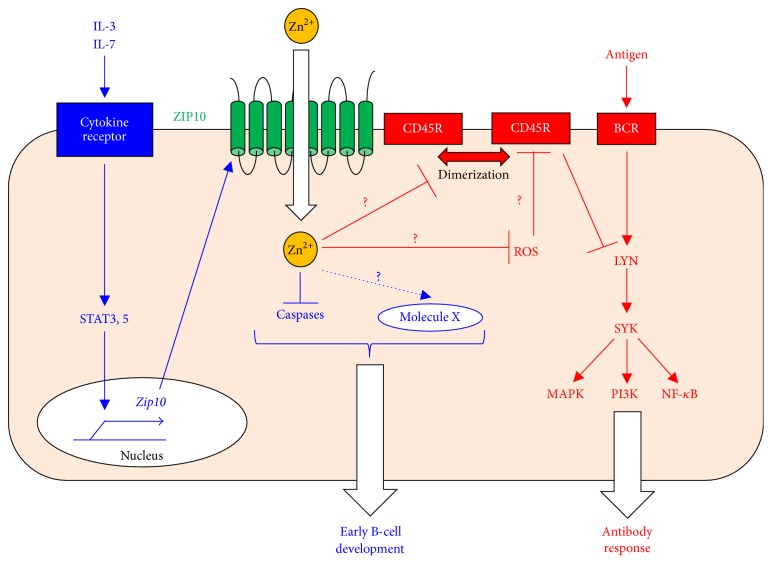
ZIP10's roles in B-cell development and function. In early B-cell development, a cytokine (1st signal) induces JAK-STAT activation (2nd signal), which is converted to an intracellular Zn signal (3rd signal) by ZIP10 upregulation. This system for converting intracellular signals promotes early B-cell survival by inhibiting caspase activation and/or by an unknown mechanism via molecule X (blue). In mature B cells, ZIP10-Zn signaling sets the threshold for the BCR signaling strength by regulating the CD45R PTPase activity (red). Thus, ZIP10 controls antibody-mediated immune responses.

BCR signaling is transmitted through multiple pathways to mediate cell activation, proliferation, and death [181–184]. BCR signaling is initiated by LYN, an SRC-family protein tyrosine kinase (Src-PTK). LYN activates SYK, which activates downstream kinases and transcription factors such as MAPK, PI3K, and NF-*κ*B [176]. Paradoxically, the overall BCR signaling is enhanced in ZIP10-deficient B cells, with hyperactivated LYN and SYK and downstream ERK, AKT, and NF-*κ*B in response to BCR stimulation [27]. This phenomenon is partly attributed to a 20% decrease in the expression and 50% decrease in the overall activity of CD45R PTPase that inhibits LYN activity (Figure 6) [185], although its precise role in regulating Src-PTKs remains controversial [186–190]. Generally, Zn has a negative impact on PTPase activity, as reported for the receptor PTPase beta [191], PTP1B [192], and SHP-1 (a negative regulator for BCR signaling) [143]. It has also been proposed that intracellular Zn is incorporated into the Golgi by ZnT5/6/7 and then released into the cytosol by ZIP9, which in turn inhibits PTPase activity to activate BCR signaling in DT40 cells [193]. If these situations are applicable to ZIP10-deficient B cells, there would be a loss of the suppressive effect of Zn normally exerted via ZIP10, resulting in reduced LYN activity due to enhanced CD45R PTPase activity. However, the opposite result is reported: downregulated CD45R PTPase activity is accompanied by enhanced LYN activation after BCR stimulation [27]. The involvement of CSK, which downregulates the LYN activity by increasing phosphorylation at its inhibitory site [194], is also unlikely, since Zn completely suppresses CSK's function [195]. In fact, when an active form of CD45R recombinant protein is coincubated with high concentration of Zn* in vitro*, its PTPase activity is suppressed [27]. However, the forced introduction of Zn into ZIP10-deficient B cells partially recovers the CD45R PTPase activity and suppresses LYN activation* ex vivo* in the stimulated cells [27]. Taken together, these results suggest that ZIP10-Zn signaling regulates the expression of CD45R while simultaneously (and indirectly) enhancing the CD45R PTPase activity through a Zn-dependent process rather than by a direct effect on PTPase activity (Figure 6).

Although the detail mechanism underlying how ZIP10-Zn signaling controls the CD45R PTPase activity* in vivo* is currently unclear, one potential target of ZIP10-Zn signaling may be an oxidant (Figure 6). Zn negatively regulates oxidants [196], which can suppress PTPase activity [197]. The involvement of reactive oxygen species (ROS) in BCR signaling as a second messenger has been reported [198, 199]. BCR engagement stimulates ROS production, which inhibits PTPase activity (e.g., SHP-1) around the BCR, thereby amplifying BCR signaling [198, 199]. Another possibility is that Zn is involved in dimerizing CD45, thereby downregulating its function (Figure 6) [200]. These issues remain to be clarified. It would be interesting to investigate (1) how ZIP10 regulates the expression of CD45R, (2) how ZIP10 regulates CD45R activity in a steady state and the BCR signaling process (e.g., whether ZIP10 forms a complex with BCR clusters), (3) which microdomains ZIP10 is located in, and (4) whether ZIP10 can act through other factors to regulate BCR signaling.

Notably, ZIP10 protein levels are low in splenic B cells and even in 293T cells with ectopic ZIP10 expression [27]. There is no difference in the intracellular Zn content of ZIP10-deficient and control B cells. Nevertheless, ZIP10 deficiency leads to a striking loss of mature B cells and a marked impairment of the antibody response. Given that a redundant system involving other Zn transporters does not appear to be active in ZIP10-deficient B cells, since there is no alteration in the expression of any other transporters, these data collectively suggest that ZIP10 is not a major contributor to the overall intracellular Zn homeostasis but rather set the threshold of BCR signal strength, probably by locally transporting small amounts of Zn from the extracellular fluid. In this regard, ZIP10 may be able to transport Zn more efficiently than other Zn transporters.

This raises the question of why, despite the augmented LYN activity, BCR-induced proliferative activity is attenuated in ZIP10-deficient B cells [27]. One possibility is that the hyperactive LYN simultaneously initiates BCR signaling and generates strong inhibitory signals mediated by FC*γ*RIIb1, CD22, and PIR-B that lead to the recruitment of the SHIP-1 and SHP-1 PTPases [201]. In fact, LYN^up/up^ mice, which express a constitutively active form of LYN, spontaneously and simultaneously activate positive (SYK) and negative (CD22, SHP-1, and SHIP-1) regulators, leading to impaired BCR-induced cell proliferation [202]. Thus, rapidly generated LYN-induced inhibitory signals may result in a signal that is insufficient for proliferation and that subsequently impairs GC formation.

It is interesting to note the similarities in immunological abnormalities in ZIP10-deficient and ZnD mice. ZnD attenuates the Th1 response, which promotes Ig CSR to noncytophilic IgG2, such as IgG2a (IgG2c in C57BL/6), without affecting the Th2 response, which promotes cytophilic IgG1 and IgE [203]. Given that ZIP10 deficiency significantly attenuates the level of IgG2c but not of IgG1 or IgE in the steady state [27], it is tempting to speculate that the loss of ZIP10 affects the signal transduction mediated by Th1 cytokines such as IFN-*γ* while that mediated by Th2 cytokines in resting B cells remains intact.

Immunological memory, which involves memory B cells and long-lived plasma cells (PCs), is primarily generated through GC reactions. Hence, it is not surprising that memory-recall responses to previously encountered antigens are attenuated in ZnD animals [74, 167, 168, 173], since a ZnD or ZIP10-deficient environment considerably attenuates GC formation [27]. These findings suggest that the impaired signaling through BCR in the mature B cells from these mice may be not sufficient to support their differentiation during GC reaction. Therefore, immunological memory cannot be properly generated in these mice. Further investigation will clarify this issue.

Early B-cell development is adversely affected by ZnD [109, 204]. Mice fed a diet marginally deficient in Zn show a 50% decline in pre-B and 25% decline in immature B-cell populations [177]. Given that steroid-implanted mice have markedly reduced numbers of pre-B and immature B cells in the bone marrow [109, 205], the effects of ZnD on early B-cell development might be partly explained by the effect of glucocorticoids, as is also the case with T cells. ZnD primarily affects B-cell precursors; resting mature B cells are relatively resistant to ZnD. Indeed, a detailed study revealed that the number of FO B cells and their levels of intracellular-free Zn were unchanged in mice fed a ZnD diet for 2 weeks [27], even though these mice clearly showed growth retardation and reduced serum Zn [27, 60]. Such an environment changes the expression levels of some Zn transporters and metallothioneins, suggesting that an unknown mechanism of resistance involving the altered expression of Zn mediators somehow maintains Zn homeostasis and avoids cell death due to insufficient Zn. However, specifically ablating ZIP10 in antigen-presenting cells causes a significant reduction in mature FO B cells [27]. Furthermore, the forced chelation of intracellular Zn by TPEN induces apoptotic cell death of mature B cells. Since the loss of ZIP10 affects neither the expression of other plasma membrane-localized Zn transporters nor the intracellular Zn content under normal conditions, these data suggest that ZIP10 plays a definitive role in mature B-cell maintenance by locally targeting the responsible molecules. In light of the enhanced overall BCR signaling already discussed, it appears that the ZIP10-deficient phenotype is a partial phenocopy of the LYN^up/up^ phenotype, since LYN^up/up^ mice also have significantly fewer resting mature B cells, probably due to enhanced signaling above a certain threshold [202]. However, LYN activation does not appear to be upregulated in ZIP10-deficient B cells in the steady state, indicating that ZIP10 maintains mature B cells through a LYN-independent mechanism [27].

ZIP10 is expressed on the surface of pro-B cells at a relatively high level compared to other bone marrow-resident B-cell subsets [28]. Specifically deleting ZIP10 in pan-B cells leads to splenic atrophy with a marked reduction of peripheral B cells due to a decreased number of pro-B cells in the bone marrow [28]. ZIP10 ablation enhances the activities of the cystein-aspartic acid proteases (caspases) 3, 8, 9, and 12 in pro-B cells, resulting in increased apoptotic cell death that can be mimicked by chemically chelating intracellular Zn using TPEN and can be restored by Zn supplementation. This finding indicates that ZIP10-Zn signaling inhibits the apoptosis induced by activated caspases and promotes pro-B-cell survival in a cell-autonomous manner (Figure 6).

JAK-STAT signaling induced by cytokine stimulation controls pro-B-cell survival and development [206] but also has oncogenic effects [207]. IL-3 or IL-7 stimulation induces ZIP10 expression through the JAK-STAT pathway [28]. In human follicular lymphoma, ZIP10 is abundant in constitutively active STAT3- and STAT5-expressing cells.* In silico* findings also show that ZIP10 is strongly expressed in acute myeloid and lymphoid leukemia. Thus, cytokine stimulation (the first signal) activates the JAK-STAT pathway (the second signal), which further induces ZIP10 expression and eventually generates ZIP10-Zn signals (the third signal). In this way, ZIP10-Zn signaling may control fate decisions in lymphocyte progenitors under physiological conditions and exacerbate malignancy under pathological conditions, according to the highly regulated pattern of ZIP10 expression [28]. Given that a rigorous selection process in early B-cell development ensures functionality and avoids autoreactivity by eliminating the majority of newly formed B cells through apoptosis [208], suppressing or augmenting the ZIP10-Zn signaling axis may lead to lymphopenia on one hand or autoimmunity and malignancy on the other. The molecular mechanism by which ZIP10-Zn signaling inhibits apoptosis is currently unclear. ZnD influences the gene expression of the BCL/BAX family [209], which is downregulated to eliminate nonreactive or autoreactive B cells during the developmental process but is also overexpressed in follicular lymphoma cells [210]. Since ZIP10-deficient pro-B cells show lower intracellular Zn level [28], ZIP10 function may be coordinated with these antiapoptotic factors.

Taken together, these findings underscore the definitive role of ZIP10-Zn signaling not only in antibody responses but also in early B-cell development and the maintenance of mature B cells (Figure 6). Collectively, these studies provide deep insights into how Zn controls lymphocyte homeostasis and function.

## 5. Conclusion

The importance of nutritional Zn for the immune systems is evident from the immunodeficiency seen in ZnD mice. Although Zn's function as a key structural or catalytic component in more than 300 enzymes and transcription factors are well known, there is a growing body of evidence that supports Zn's role as a second messenger in a variety of cellular activities. The intracellular Zn concentration can be changed by immune-related extracellular stimulation, and the subsequent crosstalk between Zn and signaling components facilitates the transduction of signaling pathways for immune homeostasis and functions. However, contradictory results have been reported regarding the effects of Zn on the immune system. Some studies show that excessive Zn impairs T-cell proliferation and cytokine production. Although the molecular mechanisms underlying the concentration-dependent effects of Zn are poorly understood, they probably involve the capacity of the intracellular buffering system to absorb large amounts of Zn and a breakdown of the system of Zn transport. Collectively, these findings strongly suggest that cellular Zn levels can determine the threshold for Zn functions in physiology and pathophysiology. In this regard, it is reasonable that Zn signaling by Zn transporters and channels would be tightly controlled in physiology.

**Figure 7:**
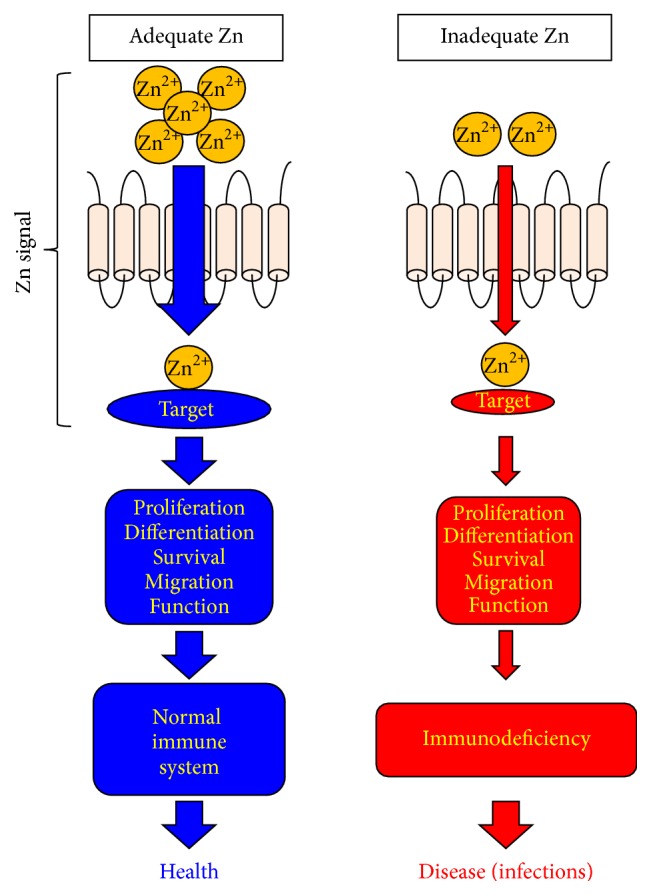
Zn-signal axes in immune system. Each Zn-signal axis targets a specific molecule and controls a variety of cellular activities such as proliferation, differentiation, survival, migration, and function via a distinct signaling pathway to control immune homeostasis and functions. ZnD (red) impairs these Zn-signal axes and leads to disease if there is no redundant machinery.

In this review, we focused mainly on the physiological effects of Zn signaling mediated by specific Zn transporter families in the adaptive immune system. Each Zn-signal axis targets a specific molecule, allowing Zn to influence a wide range of cellular activities such as proliferation, differentiation, survival, migration, and function by selectively regulating distinct signaling pathways in immune systems (Figure 7). The disruption of a Zn-signal axis by ZnD can cause immunodeficiency if there is no redundant machinery. However, there are still few studies of the involvement of the individual Zn transporters in immune homeostasis and functions; thus, research into Zn signaling in humoral immunity has barely scratched the surface. Since ZnD depresses both primary and secondary immune responses, it will be important to use both nutritional and genetic approaches to investigate Zn signaling in the prominent cell types involved in each immunological process (e.g., Tfh cells, GC B cells, and memory B and memory T cells). These analyses may provide a deeper understanding of the relevance of Zn signals in adaptive immunity and lead to novel therapeutic drugs and vaccines for immune-related disorders and infections.

## Figures and Tables

**Table 1:** Zn transporters in physiology and pathophysiology.

Gene symbol	Protein name	Mutation type	Phenotype and disorder	Reference
SLC39A1	ZIP1	KO	Abnormal embryonic development	[15]

SLC39A2	ZIP2	KO	Abnormal embryonic development	[16]

SLC39A3^*∗*1^	ZIP3	KO	Abnormal embryonic and T-cell development	[14]

SLC39A4^*∗*2^	ZIP4	KO	Embryonic lethality	[18, 20–22, 60]
		Mutation	AE	

SLC39A5	ZIP5	Mutation	Autosomal dominant nonsyndromic high myopia	[23]

SLC39A8^*∗*3^	ZIP8	Hypomorphic mutation	Impaired multiple-organ organogenesis and hematopoiesis	[24–26, 61]
			Abnormal innate immune function	
		KO	Osteoarthritis	
		SNP	Schizophrenia	

SLC39A10^*∗*4^	ZIP10	KO	Abnormal early B-cell development	[27, 28]
			Impaired humoral immune response	

SLC39A12	ZIP12	KO	Attenuation of pulmonary hypertension in a hypoxic atmosphere	[29]

SLC39A13	ZIP13	KO	Connective tissue dysplasia	[34, 62]
		Mutation	SCD-EDS	

SLC39A14	ZIP14	KO	Growth retardation and impaired gluconeogenesis	[35–38]
			Impaired hepatocyte proliferation during liver regeneration after hepatectomy	
			Decreased insulin signaling, hypertrophied adipocytes, and increased adipose cytokine production and plasma leptin	
		Mutation	Parkinsonism-dystonia and neurodegeneration with hypermanganesemia in childhood	

SLC30A1	ZnT1	KO	Embryonic lethality	[39, 63]
			Abnormal vulva formation	

SLC30A2	ZnT2	Mutation KO	Low Zn in milk	[40–43]

SLC30A3	ZnT3	KO	Prone to seizures Alzheimer's disease-like abnormalities	[45, 64]

SLC30A4	ZnT4	Mutation	Lethal milk: lm Low Zn in milk	[46]

SLC30A5	ZnT5	KO	Growth retardation, osteopenia, hypodontia, and male-specific cardiac death	[47, 48]
			Impaired mast-cell functions	

SLC30A7	ZnT7	KO	Reduced body fat accumulation	[49, 50]
			Insulin resistance, glucose intolerance, and hyperglycemia on a high-fat diet	

SLC30A8	ZnT8	KO	Type 2 diabetes mellitus	[51–56]
		SNP	Type 1 and 2 diabetes mellitus	

SLC30A10	ZnT10	Mutation	Parkinsonism, dystonia, hypermanganesemia, polycythemia, and chronic liver disease	[57–59]

^*∗*1^Mice with a targeted ZIP3 deletion show lower DP thymocyte counts but increased number of CD4^+^ SP or CD8^+^ SP thymocytes under a Zn-limiting condition.

^*∗*2^Patients with ZIP4 mutation (AE) show severe ZnD symptoms characterized by immunodeficiency with thymic atrophy and lymphopenia, and by recurrent infections. Epidermal LCs, which inhibit ICD triggered by the ATP release from epidermal keratinocytes, are significantly reduced in the lesions of AE patients, resulting in inflammatory skin manifestations. However, oral Zn supplementation allows LCs to recolonize and improve clinical symptoms in these patients.

^*∗*3^Fetal fibroblasts from ZIP8 hypomorphic mice exhibit dysregulated Zn uptake and increased NF-*κ*B activation due to insufficient control of I*κ*B kinase. Consistent with this, mice given ZnD dietary intakes develop excessive inflammation to polymicrobial sepsis.

^*∗*4^Mice with a targeted disruption of ZIP10 show impaired early B-cell development and antibody response, due to increased caspase activity and decreased CD45R PTPase activity, respectively.

## Abbreviations

ACD: Allergic contact dermatitis
AE: Acrodermatitis enteropathica
ATP: Adenosine triphosphate
BCL: B-cell lymphoma
BCR: B-cell receptor
BMP: Bone morphogenetic protein
cAMP: Cyclic adenosine monophosphate
CD: Cluster of differentiation
CIA: Collagen-induced arthritis
CN: Calcineurin
CREB: cAMP response element-binding protein
CrO: Croton oil
CSR: Class-switch recombination
CTL: Cytotoxic T lymphocyte
DAG: Diacylglycerol
DCs: Dendritic cells
DN: Double negative
DNFB: Dinitrofluorobenzene
DP: Double positive
EAE: Experimental autoimmune encephalomyelitis
EMT: Epithelial mesenchymal transition
ER: Endoplasmic reticulum
ERK: Extracellular signal-regulated kinase
Fc*ε*RI: Fc epsilon RI
FO: Follicular
GC: Germinal center
G-CSF: Granulocyte-colony stimulating factor
GPCR: G-protein coupled receptor
ICD: Irritant contact dermatitis
IFN-*γ*: Interferon gamma
Ig: Immunoglobulin
IL: Interleukin
IMM: Immature
IP3: Inositol trisphosphate
ITAM: Immunoreceptor tyrosine-based activation motif
JAK: Janus kinase
LCs: Langerhans cells
LPS: Lipopolysaccharide
LTCC: L-type calcium channel
MAPK: Mitogen-activated protein kinase
MHC-II: Major histocompatibility complex II
MZ: Marginal zone
NF-*κ*B: Nuclear factor-kappa B
PALS: Periarterial lymphatic sheath
PCs: Plasma cells
PDE: Phosphodiesterase
PFC: Plaque-forming cell
PHA: Phytohemagglutinin
PKC: Protein kinase C
PKC*θ*: Protein kinase C theta
PLC*γ*1: Phospholipase C, gamma 1
PTP1B: Protein tyrosine phosphatase 1B
PTPase: Protein tyrosine phosphatase
ROS: Reactive oxygen species
SCD-EDS: Spondylocheirodysplastic form of Ehlers-Danlos syndrome
SLC: Solute carrier family
SMAD: Sma- and Mad-related family
SP: Single positive
Src-PTKs: SRC-family protein tyrosine kinases
STAT: Signal transducers and activator of transcription
SYK: Spleen tyrosine kinase
TCR: T-cell receptor
TD: T-cell dependent
Tfh: Follicular helper T cell
TGF-*β*: Transforming growth factor beta
Th: Helper T cell
TI: T-cell independent
TLR: Toll-like receptor
TNF-*α*/*β*: Tumor necrosis factor alpha/beta
TPEN: * N,N,N,N* tetrakis (2-pyridylmethyl) ethylenediamine
Treg: Regulatory T cell
TSLP: Thymic stromal lymphopoietin
ZIP: Zrt/Irt-like protein family
ZnD: Zinc deficiency (deficient)
ZnT: Zinc transporter family.
